# Neuropsychiatric Manifestations of Thyroid Diseases

**DOI:** 10.7759/cureus.33987

**Published:** 2023-01-20

**Authors:** Vedant Lekurwale, Sourya Acharya, Samarth Shukla, Sunil Kumar

**Affiliations:** 1 Medical School, Jawaharlal Nehru Medical College, Datta Meghe Institute of Medical Sciences, Wardha, IND; 2 Internal Medicine, Jawaharlal Nehru Medical College, Datta Meghe Institute of Medical Sciences, Wardha, IND; 3 Pathology, Jawaharlal Nehru Medical College, Datta Meghe Institute of Medical Sciences, Wardha, IND

**Keywords:** mental disorders, autoimmune hashimoto encephalopathy, mania, cognitive impairment, thyroid dysfunction, hyperthyroidism, hypothyroidism, depression, dementia, thyroid

## Abstract

Thyroid disorders are known to cause neuropsychiatric manifestations. Various neuropsychiatric manifestations are depression, dementia, mania, and autoimmune Hashimoto encephalopathy. Numerous investigations carried out in the previous 50-60 years have been evaluated critically. The pathophysiology of neuropsychiatric symptoms of thyroid diseases is described in the current study and its link with autoimmune Hashimoto encephalopathy is also discussed.

Furthermore, this paper also describes the association between thyroid-stimulating hormones and cognitive impairment. Hypothyroidism is associated with depression and mania, and hyperthyroidism is linked with dementia and mania. The association between Graves' disease and various mental disorders such as depressive and anxiety disorders is also discussed. The aim of this study is to review the relationship between various neuropsychiatric disorders and thyroid diseases. A literature search from the PubMed database to find various neuropsychiatric manifestations of thyroid disorders in the adult population was conducted. According to the review of the studies, cognitive impairment can result from thyroid disease. It has not been possible to demonstrate how hyperthyroidism can hasten the process of developing dementia. However, subclinical hyperthyroidism, thyroid-stimulating hormone (TSH) levels below the normal range, and high free thyroxine (T4) levels all raise the risk of dementia in the elderly. Additionally, the potential mechanisms underlying this association have been examined. A quick summary of the research on mania as a clinical symptom of hypothyroidism and its likely causes and pathogenesis is also reviewed. There is no dearth of evidence that describes various neuropsychiatric manifestation in thyroid disorders.

## Introduction and background

The growth of the central nervous system (CNS) throughout gestation and the first few months of life are significantly influenced by thyroid hormones (TH) [1]. Numerous genes exhibit noticeable alterations in response to TH throughout these formative years. Furthermore, genes in the growing brain that respond to TH in childhood do not respond to TH in adulthood. However, thyroid adult dysfunction is frequently linked to a variety of psychiatric and cognitive issues [2-6]. The underlying mechanism of the thyroid autoimmune disease-related alterations in brain tissue is complicated but also includes changes in neuronal activity, variations through cellular metabolism and blood-brain barrier, gene expression in glial or neuronal cells, elevated risk of vascular dementia as well as cerebral inflammatory illness in conditions of thyroid autoimmune disease. Cognitive and mental illnesses are typically linked to clinical thyroid dysfunction [2-8]. Overt hypothyroidism frequently manifests as cognitive impairment, distress, and sadness, whereas hyperthyroidism can result in agitation, severe psychosis, and apathy, particularly in the elderly [7]. In the presence of hypothyroidism, reversible dementia is common.

## Review

**Figure 1:**
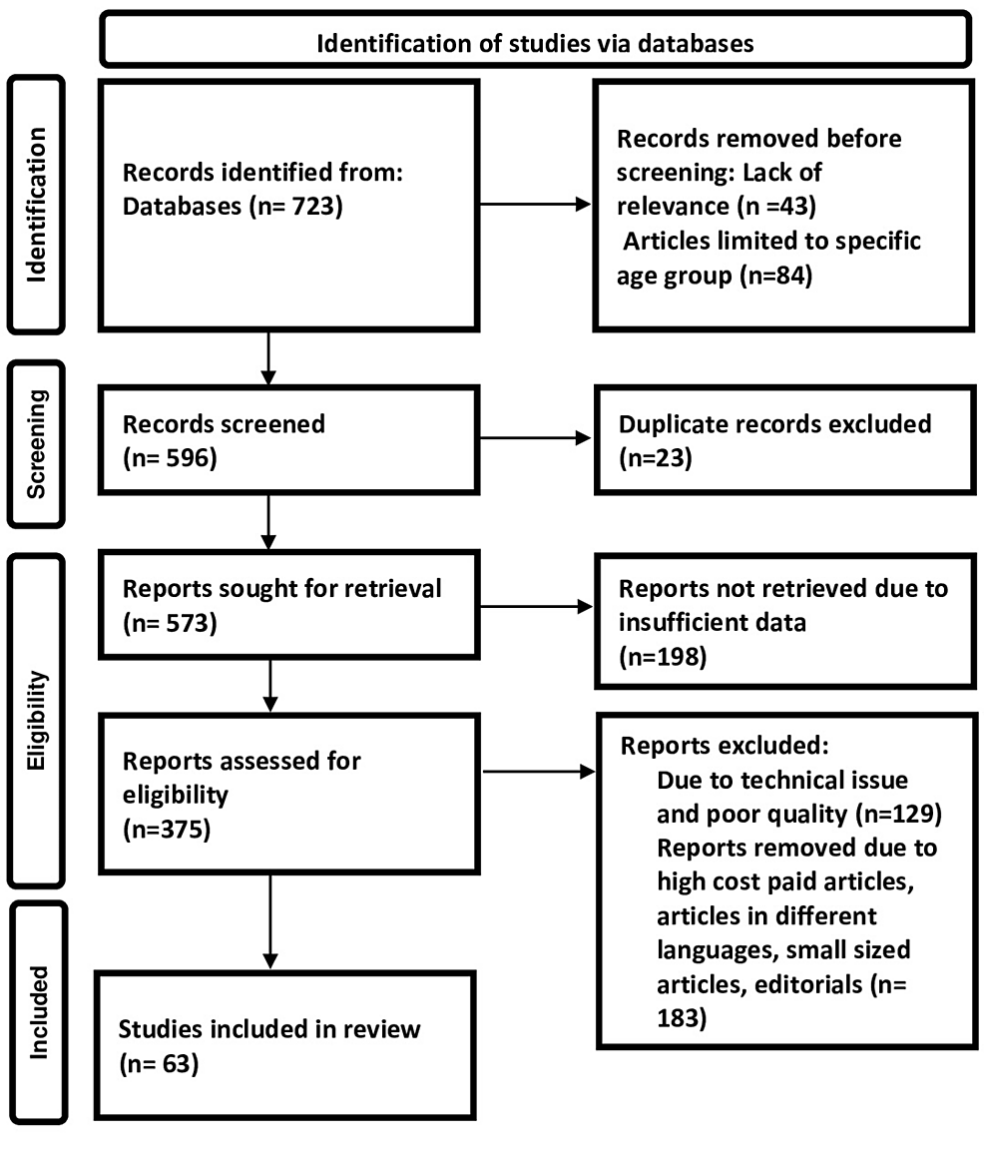
PRISMA model for search strategy. PRISMA: Preferred Reporting Items for Systemic Reviews and Meta-Analyses.

The following keywords were used to conduct searches in Embase, Scopus, Cochrane, Google Scholar, and advanced PubMed databases: mental disorders, hyperthyroidism and mania, hypothyroidism and depression, mania, and autoimmune Hashimoto encephalopathy. The search yielded 723 articles, of which 63 research publications were selected for research. The methodology of the Preferred Reporting Items for Systemic Reviews and Meta-Analyses (PRISMA) method is shown in Figure 1.

It is currently unclear if slight variations in plasma TH levels can raise the risk of cognitive deterioration. It was found that there were two types of subclinical thyroid disorders: (1) subclinical hyperthyroidism, characterized as a decrease in serum thyroid stimulating hormone (TSH) with normal levels of free thyroxine (FT4) and triiodothyronine (FT3), and (2) subclinical hypothyroidism, defined as a normal FT4 and TSH of 10 mIU/L and greater than the upper limit of the conventional recommended ranges [9]. In adults under 65, subclinical hypothyroidism has been linked to vascular dementia, and stroke [10-12]. Nonetheless, in humans over the age of 75, there doesn't appear to be much of a difference. Clinical aftereffects of subclinical hypothyroidism [13-16] with the exception of depression may be present [17]. In patients, with age more than 60, TSH levels below 10 mIU/L do not appear to substantially reduce mental function [18-20]. Being under 75 years of age was linked to a significant risk of cognitive problems in a meta-analysis of 13 studies including patients with subclinical hypothyroidism. There is some proof that thyroid conditions that are subclinically present, such as subclinical hypothyroidism or subclinical hyperthyroidism may also impair cognition, despite the fact that clinically obvious thyroid illness might have this effect. Although levothyroxine can treat dementia when there is overt hypothyroidism, levothyroxine's impact on many physiological processes during treatment is debatable regarding depressed symptoms in individuals with subclinical hypothyroidism. In people over 60 with subclinical anxiety, the risk of depression is four times higher and hypothyroidism is more likely (odds ratio: 4.886; 95% CI: 2.768-8.627) [17]. Moreover, hypothyroidism may be more common in individuals who have depression, and the presence of thyroid peroxidase antibodies is linked to depressive symptoms. In patients receiving subclinical hypothyroidism therapy with radioactive iodine (I-131) their Hamilton's Depression Rating scores were considerably higher. Usage of levothyroxine and risk factors for depression were present in people with serum TSH levels of more than 10mIU/L. The underlying genetic variation may exist in all persons and may play a secondary role in the association between severe depressive disorders and disruptions in the hypothalamic-pituitary-thyroid (HPT) axis [21]. Lifelong serious depression is connected with the rs11206244 genotype in American females. Both White and African persons have several genotypic variants including one in the 3 ′UTR of DIO1 (rs11206244) and changed FT4 levels are linked to lifelong serious depression [21]. Despite the fact that additional studies were unable to corroborate the relationship between the two variants of DIO1 with depression, genetic changes that impair thyroid function may play a role in the development of severe depression [22].

The effect of thyroid hormones T3/T4 on the cerebral tissues is shown in Table 1. Variations in the levels of T3/T4 can lead to autoimmune diseases, cerebrovascular changes, and blood-brain barrier changes. It also has an effect on neurotransmission and various metabolic activities in the body and also in gene transcription. The process of inflammation and oxidation is also affected leading to inflammatory and oxidative stress. Most importantly it has a role in psychiatric disorders. 

**Table 1:** Effect of Thyroid Hormones on Cerebral Tissues

Effect of T3/T4 on the brain		
Autoimmune disease	Cerebrovascular diseases	Blood-brain barrier changes
Neurotransmission	Oxidative stress	Metabolism
Inflammatory stress	Gene transcription	Psychiatric disorders

Association of thyroid-stimulating hormone and cognitive impairment

In middle-aged and older persons, overt hyperthyroidism as well as clinical and subclinical hypothyroidism are linked to impaired memory, alteration in reaction time, and visuospatial organization. Previous epidemiological studies have shown an affiliation between dementia and subclinical hyperthyroidism (SH) or low serum TSH values, however, its association in the elderly population is unclear [23]. A condition known as dementia is a clinical illness that is defined by a steady decline in cognitive ability and functional impairment. About 1% of people in their 60s and 70s have dementia and it rises to 45% for older people in the most advanced ages of 95 years and above. Despite the documented effects of clinical dementia, cognitive performance in older adults is complex and dependent on several factors, and the effects of thyroid diseases on cognitive function are poorly understood [23]. The connection between levels of thyroid hormone (TH) and older people's cognitive function within the typical range of references is linked to cognitive impairment. Given that thyroid hormone concentrations alter as people age increases and become less cognitive it is often accompanied by physiological changes that come with age. Changes in thyroid function could be tangentially connected to cognition as we age normally [23].

Hyperthyroidism together with dementia

The most likely cause of hyperthyroidism is toxic nodular goiter, which is accompanied by Graves' disease. Thyrotoxicosis refers to thyroid malfunction brought on by iodine or medications; intentional administration of high thyroid hormones is another frequent cause of thyrotoxicosis [24]. In patients with dementia, household and close acquaintances are also impacted by the disease's progression and spread. The three primary adverse consequences of dementia are cognitive deterioration, depression symptoms, and mania [25]. Every time an aged patient's physical or mental functioning fluctuates, hyperthyroidism must be taken into account [26]. Thyroid hormones are crucial for the normal performance of the psychological function of the brain. This article is focused on both clinical and subclinical thyroid issues to determine the causal connection between thyroid and memory loss. The leading causes of Alzheimer's disease (AD), i.e. Lewy body dementia and frontal lobe dementia, are the degradation of the brain which accounts for around 70% of classic dementia cases [27]. In a study by George et al. with 12,481 participants, it was found that hyperthyroid patients had a greater risk of dementia than euthyroid participants (hazard ratio (HR) (95% CI): 1.40 (1.02 - 1.92) [28]. Thyroid autoimmunity (AITD) and dementia (high or low) are at increased risk due to abnormal thyroid hormone levels [28]. In a research of the psychiatric community upon admittance, the prevalence of psychotic symptoms was greater in patients with positive thyroperoxidase antibody (TPO-Ab) and individuals with low blood TSH, but the incidence of other illnesses like schizophrenia or some other mental health issue did not change [29].

A study by Döbert et al. investigated the connection between both thyroid antibodies and thyrotropin (TSH) and different types of dementia. Individuals with low or questionable TSH levels were shown to have a greater risk of acquiring dementia, especially vascular dementia [30]. A cross-sectional hospital-based investigation was conducted by Agarwal et al. on patients diagnosed with AD/vascular dementia (VaD) (114 Alzheimer's disease patients with a mean age of 65 and 35 VaD patients with a mean age of 62 years). Subclinical AD and hyperthyroidism were associated significantly according to routine testing of free T3, free T4, and TSH tests on 105 control volunteers with a standard age of 62 years [31]. As per research with a greater sample size, there is statistically compelling evidence that hyperthyroidism increases the likelihood of dementia. Cognitive decline is linked with numerous thyroid conditions, involving hypothyroidism, hyperthyroidism, and autoimmune thyroid diseases. The frequency of dementia is increased in hyperthyroid diseases when compared to euthyroidism. The majority of studies have discovered evidence to back up the hypothesis that increased thyroid hormone levels raise the risk of memory loss. The amyloid-beta protein precursor (APP) gene's expression is regulated by thyroid hormone, according to research by Belandia et al. [32,33]. Furthermore, in both in vitro and in vivo models, O'Barr et al. discovered that thyroid hormone affects endogenous amyloid-beta precursor protein gene production and processing [34]. It is now known that vascular risk factors and cardiovascular illness increase the likelihood of developing AD [35,36], hence it is conceivable to find proof that thyroid dysfunction causes dementia through an inherent vascular mechanism. Moreover, cardiovascular disease prevalence is increased by both clinical and sub-clinical thyroid dysfunction [37-39]. The type 1 and type 2 deiodinase gene polymorphisms and iodothyronine levels in the aged persons were found to be correlated in the Rotterdam Study by De Jong et al [40]. Patients with hyperthyroidism were shown to have lower antioxidant metabolites and oxidative stress. Low levels of thyroid hormone cause APP to express more, which boosts amyloid peptide and amyloid levels because of which there is thyroid hormone activation and neuroserpins upregulation and there is less amyloid-beta plaque clearance and eradication from the brain. Oxidative stress and a drop in the level of antioxidant metabolites in hyperthyroid people cause neurodegeneration in the brain and neuronal death as stated in the study by Bianchi et al. (2020). Dysfunction of thyroid-related vascular risk factors that lead to AD may be the cause of the brain's degenerative alterations [41]. The overall quantity of tau protein was greater in hyperthyroid patients [42].

Hyperthyroidism and mania

One of the most frequent causes of thyrotoxicosis is Graves' disease. The majority of patients suffering from Graves' disease exhibit the traditional signs and symptoms of hyperthyroidism [43]. The way Graves' disease manifests itself depends on the severity of the condition, period of hyperthyroidism, sex of the individual, and other coexisting medical conditions [43]. Weight reduction, weariness, excessive sweating, shaking, tachycardia, nervousness, sleep disturbance, and perspiration are the most typical symptoms of Graves' disease. Individuals infrequently could exhibit psychosis, mania, or a mix of the two as their initial presenting symptoms [44-46]. States of thyrotoxicosis have been linked to psychiatric disorders. With the introduction of anti-thyroid medications, this appearance is becoming less common. Thyrotoxicosis and hypothyroid states are linked to Basedow psychosis and myxedema madness respectively. A patient with Graves' disease will experience irritability and hyperexcitability symptoms, but they rarely match the standards for a mania diagnosis. It is uncertain what causes thyrotoxicosis-related behavioral disturbances [47]. The hypothesis is that the hyperthyroid-induced hyperadrenergic system interferes with the adrenergic pathway which unites the frontal lobe to the locus coeruleus, which controls concentration and alertness [48]. Mania, psychosis, or a mix of the two may be Graves' disease manifestations. Therapeutic interventions include beta-blockers and anti-thyroid medications with treatment, symptoms ought to bring out improvements in the patients.

Hypothyroidism and depression

It has long been believed that there is a connection between hypothyroidism and depression, but it is difficult to pinpoint exactly what is the connection. The relationship between thyroid function and depression is still not well understood, despite the great number of studies. Although the specifics of this relationship and what causes it have not been conclusively demonstrated, it is widely believed and taught in medicine that hypothyroidism and depression are related. There are similarities in symptoms between people with hypothyroidism and those who are profoundly depressed. The therapeutic use of thyroid hormones in the treatment of depression supports this statement. The apparent anomalies in the hypothalamic-pituitary-thyroid axis of depressed individuals are also seen. Thyroid hormones affect noradrenergic and serotonergic neurotransmission, which are targets for current antidepressant medications [49-53] and play a significant role in the etiology of depression.

Association of hypothyroidism with mania and depression

Numerous neuropsychiatric symptoms of hypothyroidism include depression, apathy, cognitive impairment, psychosis, and affective disorders [54]. The Clinical Society of London's Committee on Myxoedema made the initial connection between primary hypothyroidism and psychosis in 1888 [55]. This condition is a prevalent clinical issue with a variety of physical manifestations. In his report on the subject from 1949, Asher described "myxoedema madness," where severe hypothyroidism was linked to acute psychosis [56]. Forgetfulness, mental sluggishness, lethargy, and emotional lability are common signs of psychological dysfunction in hypothyroidism. It's usual to have cognitive changes that affect one's ability to pay attention, concentrate, perceive, and think quickly. The most frequent emotional condition seen in these people is depression. Howland discovered in his meta-analysis that subclinical hypothyroidism affects about 50% of people with resistant depression [56]. Patients who suffer from depression account for up to 20% of those with detectable antithyroid antibodies [57]. People with advanced diseases often experience suicidal thoughts, delusions, and hallucinations. Mania is frequently observed in conditions of hyperthyroidism, although certain case reports also note the occurrence of the same condition in severe hypothyroidism [58].

Mental disorders in association with Grave's disease

Thyroid disorders and mental illness are often interrelated. T3 has been intimately associated with depression and anxiety due to its regulatory effects on serotonin and nor-adrenaline.

Influence of thyroid hormones on mood

In studies on population, it has been shown that a variety of psychosocial factors which includes traumas, life events, everyday pressures, social support, and different personality qualities have an impact on the incidence and exacerbation of mental disorders. From a scientific standpoint, the monoamine hypothesis [59], which connects mental illnesses to the action of monoamine neurotransmitters, is the most compelling explanation. In relation to thyroid hormones, triiodothyronine (T3) is known to control the levels and activities of serotonin and noradrenaline [60]. Depression and anxiety disorders can be brought on by low T3, and these illnesses are also associated with low serotonin and noradrenaline levels. According to a meta-analysis, 25% of cases with resistant depression were successfully treated when T3 was added to tricyclic antidepressants [61]. Serotonin and noradrenaline levels rise as a result of T3's effects. Throughout episodes of depression, the hypothalamus releases greater thyrotropin-releasing hormone (TRH), which raises serotonin levels in the brain. The reason for this is that serotonin suppresses TRH, which in turn leads the pituitary to release thyrotropins, which in turn induces the thyroid to generate thyroxine (T4) and T3. GD commonly coexists with disorders including depression, anxiety, and mental disease. Both biological influences, such as the impact of thyroid hormones, and psychological factors such as stress and disease awareness, may affect how quickly an illness will progress. Patients with GD and hyperthyroidism may benefit from antipsychotic medications and psychotherapies based on the bio-psycho-social medical model.

Autoimmune Hashimoto encephalopathy

Increased levels of thyroid autoantibodies in the serum and cerebrospinal fluid (CSF) are the hallmarks of an uncommon neuropsychiatric condition known as autoimmune Hashimoto's encephalopathy, which is extremely sensitive to glucocorticoid therapy [62]. Elevated thyroid autoantibody levels in the blood and cerebrospinal fluid (CSF), which are very susceptible to glucocorticoid treatment, are a defining feature of autoimmune Hashimoto encephalopathy, a rare neuropsychiatric illness [62]. Around 85% of the cases are of females aged between 40 to 50 years; however, men often manifest more serious neuropsychiatric symptoms [63]. Clinical signs of Hashimoto encephalopathy might range from headaches to personality changes, dementia, delusional behavior, seizures, ataxia, aphasia, and even coma, and hallucinations [63]. There are typically two types of Hashimoto encephalopathy distinguished by certain tendencies; one is a vasculitic syndrome that "resembles a stroke" and recurs frequently, and the other is a silent, slowly progressing cognitive and psychotic disorder with localized symptoms [63]. The neuropsychiatric disorders linked to thyroid dysfunction are a result of alterations in neurotransmission, brain metabolism, and blood-brain barrier (BBB) function brought on by thyroid hormones.

## Conclusions

Thyroid diseases can present with various neuropsychiatric manifestations with or without any overt clinical signs along the temporal course of illness. So while dealing with neurocognitive/neuropsychiatric manifestations, thyroid disorder should be ruled out by appropriate thyroid function tests which include TSH, free T3, free T4, reverse T3, and thyroid antibody tests.

## References

[1] Bernal J (2017). Thyroid hormone regulated genes in cerebral cortex development. J Endocrinol.

[2] Joffe RT, Sokolov ST (1994). Thyroid hormones, the brain, and affective disorders. Crit Rev Neurobiol.

[3] Bauer M, Goetz T, Glenn T, Whybrow PC (2008). The thyroid-brain interaction in thyroid disorders and mood disorders. J Neuroendocrinol.

[4] Wu Y, Pei Y, Wang F, Xu D, Cui W (2016). Higher FT4 or TSH below the normal range are associated with increased risk of dementia: a meta-analysis of 11 studies. Sci Rep.

[5] Winkler A, Weimar C, Jöckel KH (2016). Thyroid-stimulating hormone and mild cognitive impairment: results of the Heinz Nixdorf Recall study. J Alzheimers Dis.

[6] Correia N, Mullally S, Cooke G (2009). Evidence for a specific defect in hippocampal memory in overt and subclinical hypothyroidism. J Clin Endocrinol Metab.

[7] Feldman AZ, Shrestha RT, Hennessey JV (2013). Neuropsychiatric manifestations of thyroid disease. Endocrinol Metab Clin North Am.

[8] Duntas LH, Maillis A (2013). Hypothyroidism and depression: salient aspects of pathogenesis and management. Minerva Endocrinol.

[9] Forti P, Olivelli V, Rietti E (2012). Serum thyroid-stimulating hormone as a predictor of cognitive impairment in an elderly cohort. Gerontology.

[10] Chaker L, Baumgartner C, den Elzen WP (2015). Subclinical hypothyroidism and the risk of stroke events and fatal stroke: an individual participant data analysis. J Clin Endocrinol Metab.

[11] Razvi S, Shakoor A, Vanderpump M, Weaver JU, Pearce SH (2008). The influence of age on the relationship between subclinical hypothyroidism and ischemic heart disease: a metaanalysis. J Clin Endocrinol Metab.

[12] Ochs N, Auer R, Bauer DC, Nanchen D, Gussekloo J, Cornuz J, Rodondi N (2008). Meta-analysis: subclinical thyroid dysfunction and the risk for coronary heart disease and mortality. Ann Intern Med.

[13] Pasqualetti G, Pagano G, Rengo G, Ferrara N, Monzani F (2015). Subclinical hypothyroidism and cognitive impairment: systematic review and meta-analysis. J Clin Endocrinol Metab.

[14] Ojala AK, Schalin-Jäntti C, Pitkälä KH, Tilvis RS, Strandberg TE (2016). Serum thyroid-stimulating hormone and cognition in older people. Age Ageing.

[15] Gussekloo J, van Exel E, de Craen AJ, Meinders AE, Frölich M, Westendorp RG (2004). Thyroid status, disability and cognitive function, and survival in old age. JAMA.

[16] Cappola AR, Fried LP, Arnold AM (2006). Thyroid status, cardiovascular risk, and mortality in older adults. JAMA.

[17] Chueire VB, Romaldini JH, Ward LS (2007). Subclinical hypothyroidism increases the risk for depression in the elderly. Arch Gerontol Geriatr.

[18] St John JA, Henderson VW, Gatto NM, McCleary CA, Spencer CA, Hodis HN, Mack WJ (2009). Mildly elevated TSH and cognition in middle-aged and older adults. Thyroid.

[19] Jorde R, Waterloo K, Storhaug H, Nyrnes A, Sundsfjord J, Jenssen TG (2006). Neuropsychological function and symptoms in subjects with subclinical hypothyroidism and the effect of thyroxine treatment. J Clin Endocrinol Metab.

[20] Roberts LM, Pattison H, Roalfe A, Franklyn J, Wilson S, Hobbs FD, Parle JV (2006). Is subclinical thyroid dysfunction in the elderly associated with depression or cognitive dysfunction?. Ann Intern Med.

[21] Philibert RA, Beach SR, Gunter TD (2011). The relationship of deiodinase 1 genotype and thyroid function to lifetime history of major depression in three independent populations. Am J Med Genet B Neuropsychiatr Genet.

[22] Gałecka E, Talarowska M, Maes M, Su KP, Górski P, Szemraj J (2016). Polymorphisms of iodothyronine deiodinases (DIO1, DIO3) genes are not associated with recurrent depressive disorder. Pharmacol Rep.

[23] Kirkegaard C, Faber J (1998). The role of thyroid hormones in depression. Eur J Endocrinol.

[24] De Leo S, Lee SY, Braverman LE (2016). Hyperthyroidism. Lancet.

[25] Volicer L (2018). Behavioral problems and dementia. Clin Geriatr Med.

[26] Hogervorst E, Huppert F, Matthews FE, Brayne C (2008). Thyroid function and cognitive decline in the MRC Cognitive Function and Ageing Study. Psychoneuroendocrinology.

[27] Brookmeyer R, Gray S, Kawas C (1998). Projections of Alzheimer's disease in the United States and the public health impact of delaying disease onset. Am J Public Health.

[28] George KM, Lutsey PL, Selvin E, Palta P, Windham BG, Folsom AR (2019). Association between thyroid dysfunction and incident dementia in the Atherosclerosis Risk in Communities Neurocognitive study. J Endocrinol Metab.

[29] Oomen HA, Schipperijn AJ, Drexhage HA (1996). The prevalence of affective disorder and in particular of a rapid cycling of bipolar disorder in patients with abnormal thyroid function tests. Clin Endocrinol (Oxf).

[30] Döbert N, Hamscho N, Menzel C (2003). Subclinical hyperthyroidism in dementia and correlation of the metabolic index in FDG-PET. Acta Med Austriaca.

[31] Agarwal R, Kushwaha S, Chhillar N, Kumar A, Dubey DK, Tripathi CB (2013). A cross-sectional study on thyroid status in North Indian elderly outpatients with dementia. Ann Indian Acad Neurol.

[32] Belandia B, Latasa MJ, Villa A, Pascual A (1998). Thyroid hormone negatively regulates the transcriptional activity of the beta-amyloid precursor protein gene. J Biol Chem.

[33] Latasa MJ, Belandia B, Pascual A (1998). Thyroid hormones regulate beta-amyloid gene splicing and protein secretion in neuroblastoma cells. Endocrinology.

[34] O'Barr SA, Oh JS, Ma C, Brent GA, Schultz JJ (2006). Thyroid hormone regulates endogenous amyloid-beta precursor protein gene expression and processing in both in vitro and in vivo models. Thyroid.

[35] Luchsinger JA, Reitz C, Honig LS, Tang MX, Shea S, Mayeux R (2005). Aggregation of vascular risk factors and risk of incident Alzheimer disease. Neurology.

[36] Newman AB, Fitzpatrick AL, Lopez O (2005). Dementia and Alzheimer's disease incidence in relationship to cardiovascular disease in the Cardiovascular Health Study cohort. J Am Geriatr Soc.

[37] Walsh JP, Bremner AP, Bulsara MK, O'Leary P, Leedman PJ, Feddema P, Michelangeli V (2005). Subclinical thyroid dysfunction as a risk factor for cardiovascular disease. Arch Intern Med.

[38] Hak AE, Pols HA, Visser TJ, Drexhage HA, Hofman A, Witteman JC (2000). Subclinical hypothyroidism is an independent risk factor for atherosclerosis and myocardial infarction in elderly women: the Rotterdam Study. Ann Intern Med.

[39] Toft AD, Boon NA (2000). Thyroid disease and the heart. Heart.

[40] de Jong FJ, Peeters RP, den Heijer T (2007). The association of polymorphisms in the type 1 and 2 deiodinase genes with circulating thyroid hormone parameters and atrophy of the medial temporal lobe. J Clin Endocrinol Metab.

[41] Bianchi G, Solaroli E, Zaccheroni V, Grossi G, Bargossi AM, Melchionda N, Marchesini G (1999). Oxidative stress and anti-oxidant metabolites in patients with hyperthyroidism: effect of treatment. Horm Metab Res.

[42] Joy Mathew C, Jose MT, Elshaikh AO, Shah L, Lee R, Cancarevic I (2020). Is hyperthyroidism a possible etiology of early onset dementia?. Cureus.

[43] Asif H, Nwachukwu I, Khan A, Rodriguez G, Bahtiyar G (2022). Hyperthyroidism presenting with mania and psychosis: a case report. Cureus.

[44] Kahaly GJ, Olivo PD (2017). Graves’ disease. N Engl J Med.

[45] Ogah OS, Timeyin AO, Kayode OA, Otukoya AS, Akinyemi RO, Adeyemi FI (2009). Graves' disease presenting as paranoid schizophrenia in a Nigerian woman: a case report. Cases J.

[46] Prabhu H, Jagdish TK, Valdiya PS, Narayana PL (1994). Graves disease with organic mood syndrome. Med J Armed Forces India.

[47] Bennett B, Mansingh A, Fenton C, Katz J (2021). Graves' disease presenting with hypomania and paranoia to the acute psychiatry service. BMJ Case Rep.

[48] Fukui T, Hasegawa Y, Takenaka H (2001). Hyperthyroid dementia: clinicoradiological findings and response to treatment. J Neurol Sci.

[49] Henley WN, Koehnle TJ (1997). Thyroid hormones and the treatment of depression: an examination of basic hormonal actions in the mature mammalian brain. Synapse.

[50] Bauer M, Heinz A, Whybrow PC (2002). Thyroid hormones, serotonin and mood: of synergy and significance in the adult brain. Mol Psychiatry.

[51] Whybrow PC, Prange AJ Jr (1981). A hypothesis of thyroid-catecholamine-receptor interaction. Its relevance to affective illness. Arch Gen Psychiatry.

[52] Gordon JT, Kaminski DM, Rozanov CB, Dratman MB (1999). Evidence that 3,3',5-triiodothyronine is concentrated in and delivered from the locus coeruleus to its noradrenergic targets via anterograde axonal transport. Neuroscience.

[53] Mason GA, Bondy SC, Nemeroff CB, Walker CH, Prange AJ Jr (1987). The effects of thyroid state on beta-adrenergic and serotonergic receptors in rat brain. Psychoneuroendocrinology.

[54] Samuels MH (2014). Psychiatric and cognitive manifestations of hypothyroidism. Curr Opin Endocrinol Diabetes Obes.

[55] Asher R (1949). Myxoedematous madness. Br Med J.

[56] Howland RH (1993). Thyroid dysfunction in refractory depression: implications for pathophysiology and treatment. J Clin Psychiatry.

[57] Nemeroff CB, Simon JS, Haggerty JJ Jr, Evans DL (1985). Antithyroid antibodies in depressed patients. Am J Psychiatry.

[58] Sathya A, Radhika R, Mahadevan S, Sriram U (2009). Mania as a presentation of primary hypothyroidism. Singapore Med J.

[59] Den Boer JA (2006). Looking beyond the monoamine hypothesis. Eur Neurol Rev.

[60] Mason GA, Walker CH, Prange AJ Jr (1993). L-triiodothyronine: is this peripheral hormone a central neurotransmitter?. Neuropsychopharmacology.

[61] Aronson R, Offman HJ, Joffe RT, Naylor CD (1996). Triiodothyronine augmentation in the treatment of refractory depression. A meta-analysis. Arch Gen Psychiatry.

[62] Leyhe T, Müssig K (2014). Cognitive and affective dysfunctions in autoimmune thyroiditis. Brain Behav Immun.

[63] Churilov LP, Sobolevskaia PA, Stroev YI (2019). Thyroid gland and brain: Enigma of Hashimoto's encephalopathy. Best Pract Res Clin Endocrinol Metab.

